# Susac's Syndrome in a Patient Diagnosed with MS for 20 Years: A Case Report

**DOI:** 10.1155/2014/214648

**Published:** 2014-02-02

**Authors:** Bijen Nazliel, Asli Akyol, Hale Zeynep Batur Caglayan, Irem Yildirim-Capraz, Ceyla Irkec

**Affiliations:** Department of Neurology, Faculty of Medicine, Gazi University, 06500 Ankara, Turkey

## Abstract

Susac's syndrome is an uncommon neurologic disorder of unknown cause. It has been described as a clinical triad of encephalopathy, hearing loss, and branch retinal artery occlusions. Clinically the diagnosis is difficult when the patient presents only a portion of a triad. We present a case with vision loss and sensorineural deafness and who had been diagnosed with MS for 20 years. Susac's syndrome is presumed to be an autoimmune endotheliopathy. Neurologic symptoms and signs are diffuse and multifocal, acute or subacute in onset, and progress during the active phase of the disease. In some patients the onset was stroke like and in others that of subacute dementia. Headache, often with migrainous features, was a prominent feature initially in more than one half of the patients. A high index of suspicion leading to correct diagnosis and early appropriate therapy may reduce the permanent sequel seen with this disease. Misdiagnosis is common. In patients in whom diagnosis and treatment are delayed permanent morbidity is higher in terms of visual loss, hearing loss, and neurologic debility. In patients in whom rapid diagnosis has led to early administration of immunosuppressive therapy, recovery can be almost complete.

## 1. Introduction

Susac's syndrome (SS) is an uncommon neurologic disorder of unknown cause. It has been described as a clinical triad of encephalopathy, hearing loss, and branch retinal artery occlusions. Clinically the diagnosis is difficult when the patient presents only a portion of a triad [1].

We present a case with vision loss and sensorineural deafness and who had been diagnosed with MS for 20 years.

## 2. Case Report 

**Figure 1:**
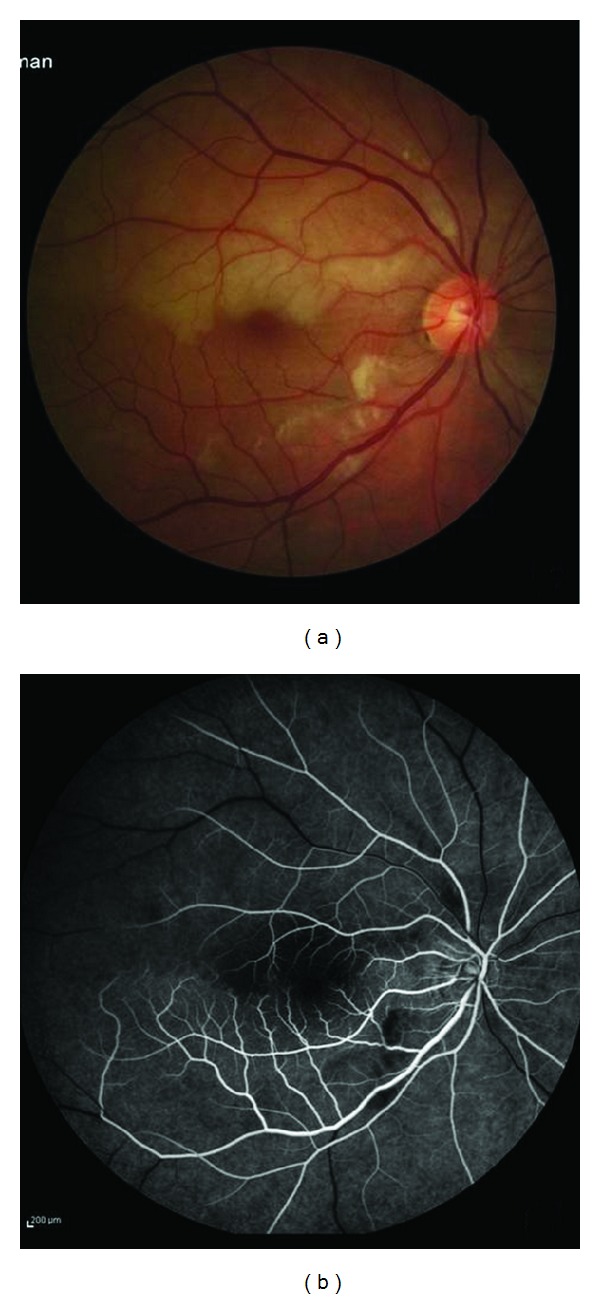
(a) Fundus photograph of the right eye with superior hemiretinal edema due to superotemporal branch retinal artery occlusion. (b) Fluorescein angiogram of the right eye revealed the presence of delayed filling of the superotemporal branch retinal artery.

A 41-year-old male patient was admitted to our outpatient clinic with a 20-year diagnosis of MS. On ophthalmic examination the visual acuity in the left eye was 20/100 and 20/20 in the right eye. No other neurological deficit or abnormality was identified in the neurological examination. In his past medical history, he had loss of vision in the left eye 20 years earlier, which did not improve at that time. He was diagnosed with MS and had received, once per month, IV 1000 mg methylprednisolone treatment for a couple of years. Seven years ago, he had sudden deafness in the left ear. An audiogram performed at that time showed total sensory neural deafness in the left and 20% loss of hearing in the right ear. Brain MRI findings were interpreted as consistent with a demyelinating disease. One year ago, he had sudden loss of vision in his right eye. A fluorescein angiogram performed at that time showed branch retinal artery occlusion in the right eye (Figure 1).

His pattern-shift visual evoked potential (PVEP) recordings at that time showed a prominent positive wave (P100) with a peak latency of 114 ms (*n*: 90–120 ms) in the right eye, while no wave form could be obtained from the left eye. He was treated with hyperbaric oxygen and 100 mg aspirin and he stated that his vision had returned to nearly normal within 20 days.

**Figure 2:**
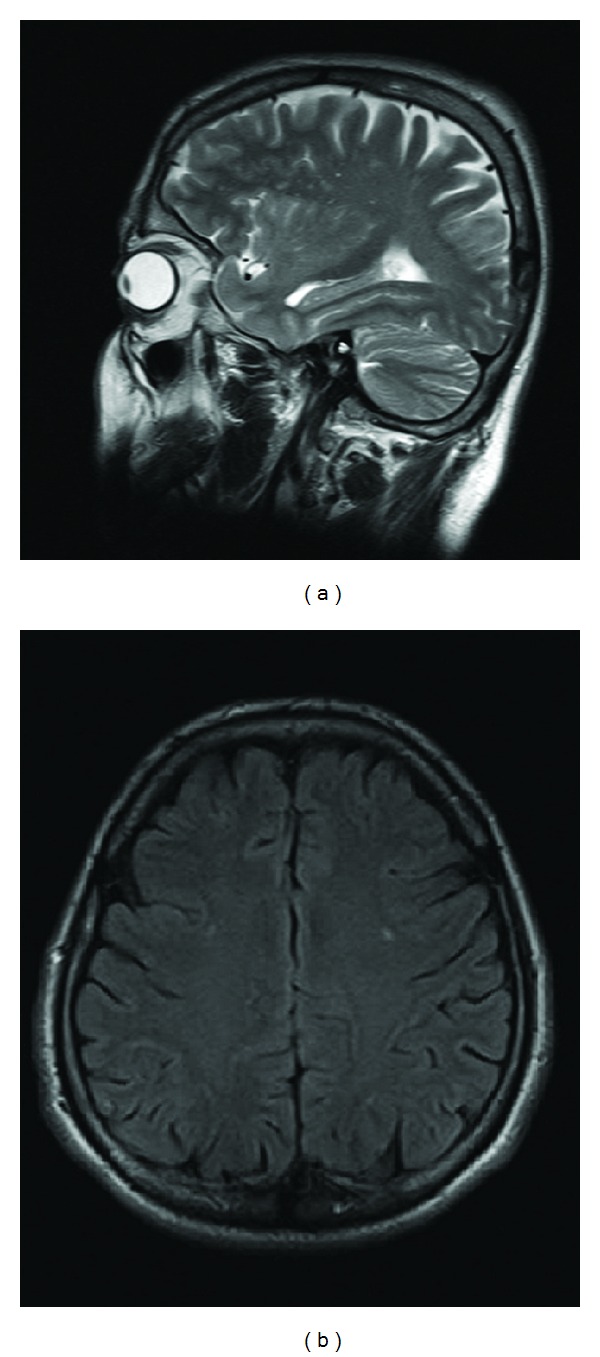
(a) T2-weighted sagittal and (b) axial flair images revealed the presence of small hyperintense pericallosal lesions and supraventricular white matter lesions.

From the evidence in his past medical history, we reevaluated the patient and reached a diagnosis of Susac's syndrome. T2-weighted brain magnetic resonance imaging (MRI) demonstrated multiple small foci of increased signal intensities (Figure 2).

**Figure 3:**
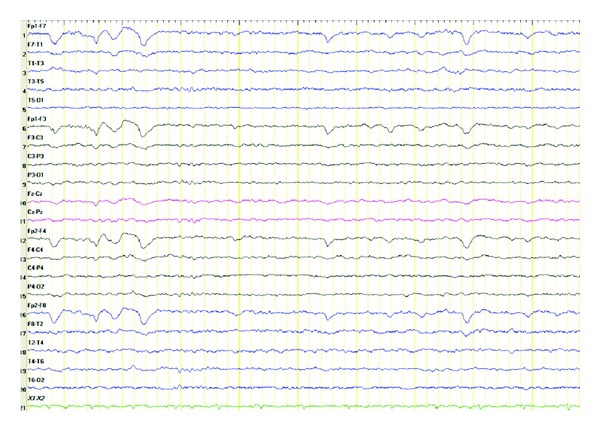
Mild diffuse slowing of the cerebral bioelectrical activity. Background activity was characterized by low voltage slow alpha rhythm (7-8 Hz) with superimposed 4–6 Hz rare theta activity especially in frontotemporal regions.

An electroencephalography (EEG) showed mild diffuse slowing of the cerebral bioelectrical activity. Background activity was characterized by low voltage slow alpha rhythm (7-8 Hz) with superimposed 4–6 Hz rare theta activity especially in frontotemporal regions (Figure 3).

In brain stem auditory evoked potential (BAEP) testing, no waveform was obtained from the left ear, while I, II, and IV waves with normal peak and interpeak latencies were present on the right. Control VEP 1 year after the event on the right eye showed that the latency of P100 in the right eye had declined from 114 ms to 102 ms. Cranial MR angiography and transthoracic and transesophageal echocardiography were normal.

ANA was weakly positive with high RF. Hematological and biochemical evaluations, including ferritin, vitamin B12, and homocysteine, were normal. Platelet count, prothrombin time, partial thromboplastin time, fibrinogen, D-dimer, and sedimentation rate were within normal limits. ACA, AFA, LA, and ds DNA were negative. Antiphospholipid antibodies, C-reactive protein, total IgA, IgM, IgE, C3, C4, and a hepatitis screen were all normal.

He had not received any treatment for Susac's syndrome over the last 20 years and had had a total of three attacks (two from the eyes, one from the ears). We questioned him again regarding neuropsychiatric symptoms. He had never had any neuropsychiatric symptom although his EEG revealed the presence of bilateral frontotemporal slowing. A number of patients who had what appears to be a partial form of the syndrome, for example, cochlear and retinal involvement without cerebral symptoms [2–5] as in our patient or cerebral and retinal involvement without hearing loss [6] or retinal involvement alone [2], have been reported. It has been stated that MRI findings may be abnormal even in the absence of symptoms specifically referable to the brain in patients with these partial forms [5, 7].

## 3. Discussion

Susac's syndrome was first described in 1979 [1]. The disease is also known as (a)-RED-M which stands for retinopathy, encephalopathy, and deafness associated microangiopathy, (b) SICRET (small infarct of cochlear, retinal, and encephalic tissues), and (c) retinocochleocerebral vasculopathy [8].

Although SS occurs in young women between the ages of 20 and 40, it may afflict men. The age range in both sexes is from 16 to 58 years, and the male to female ratio is 3 : 1 [9]. Affected patients have multiple branch retinal occlusions that typically are bilateral, progressive hearing loss, and various neurologic presentations including but not limited to psychiatric changes and encephalopathy [10]. The branch retinal artery occlusion may be quite extensive and spectacular or may be very subtle [11]. Arteriolar occlusions usually are caused by emboli that typically are fleeting in nature [10]. If the infarctions are extensive and involve the posterior pole, the patient will complain of impaired vision. If the occlusions occur in the more peripheral portion of the retina or if the patient is encephalopathic, he may not describe visual symptoms. The branch retinal artery occlusions are invariably bilateral and may be the presenting symptoms of the illness or occur later in the clinical course [11].

Hearing loss can be a dramatic and severely disabilating feature of SS. It often occurs overnight and may affect both ears. A loss of low or middle frequencies is typical, but loss of high frequencies can also occur [12]. The predominance of low and medium tone loss is suggestive of cochlear apical damage caused by occlusions of the cochlear end arterioles. Loss of high-frequency tone is a marker of more significant cochlear damage [10]. The hearing loss is often accompanied by vertigo and tinnitus [12]. The cranial nerves are not involved in Susac's syndrome. The hearing loss is due to cochlear involvement and vertigo if present is due to semicircular canal involvement [13].

Neurologic symptoms and signs are diffuse and multifocal, acute or subacute in onset, and progress during the active phase of the disease. In some patients the onset was stroke like and in others that of subacute dementia. Headache, often with migrainous features, was a prominent feature initially in more than one half of the patients. Most patients have abnormalities in cognition, memory, behavior and affect including pseudobulbar behavior and abulia sometimes with bizarre neuropsychiatric manifestations [7]. The encephalopathy may progress to a stage where the patient is totally unable to communicate. Seizures and myoclonus may occur [11].

Susac's syndrome is presumed to be an autoimmune endotheliopathy. In biopsies of the brain, microinfarcts with loss of neurons, axons, and myelin in the white and grey matter could be detected. The microinfarcts are caused by microangiopathic process with arteriole wall proliferation, lymphocyte infiltration, destruction of the capillary network, and basal lamina thickening [12]. Diagnosis and management often require a multidisciplinary effort involving a neurologist, neuroopthalmologist, otolaryngologist, neuroradiologist, and rheumatologist [14]. Brain magnetic resonance (MR) imaging plays an important role in evaluation of patients suspected of having SS [8]. On MRI, the typical lesions are small multifocal lesions of 3–7 mm. The most important diagnostic sign and very typical for SS is the snowball-like lesions in the center of the corpus callosum. At the onset of the disease, before treatment contrast enhancement around small vessels representing a perivascular leakage and leptomeningeal contrast enhancement can be seen. Other typical MRI findings are a series of small central holes that gives the corpus callosum a riddled aspect. The snowballs result in residual holes, especially in the splenium of the c. callosum. The linear defect of the corpus callosum reflects microinfarct of obliquely radiating axons, the so called “spokes” or wedged shaped lesions extending from the corpus callosum, the so called “icicles.” The most important differential diagnoses are multiple sclerosis (MS) and acute disseminated encephalomyelitis (ADEM). In MS and ADEM, the lesions are on the undersurface and at the septal interface of the corpus callosum. In SS, the lesions are lying on the center of the corpus callosum and are sparing the periphery of it. In ADEM and MS leptomeningeal enhancement is uncommon. MS has typically ovoid lesions whereas the lesions in SS are round and resemble snowballs. MS is a disease predominantly involving the white matter whereas deep gray matter involvement is a common finding in SS [12]. Spinal fluid examination has invariably shown an elevated protein in the range of 100 to 790 mg/dL and minimal pleocytosis (5–15 lymphocytes) occurs occasionally [11]. EEG shows diffuse slowing [15] while cerebral arteriography findings are almost normal, because the involved precapillary arterioles (<100 *μ*m) are beyond the resolution of arteriography. Fluorescein angiography however is extremely useful and will often show the branch retinal artery occlusion as well as the pathognomonic multifocal fluorescence of the branch arterioles [13].

The classical triad is pathognomonic for SS, but the three elements are not always present at the same time [1]. One or two of the classical triad may be absent symptomatically and/or be difficult to detect clinically, especially at presentation but often beyond [16]. Patients may present with isolated encephalopathy, unexplained visual disturbance, or hearing loss and go undiagnosed until a full triad is assumed [14]. Based on the hypothesis of it being an autoimmune disease, treatment has to be immunosuppressive. Considering the often severe residual deficits of brain, eye, and ear functions in these mainly young patients, treatment has to be significantly aggressive to prevent further damage and relapses [12]. Treatment with IV methylprednisolone followed by oral steroids is recommended [13]. Intravenous immunoglobulin (IV IG) can be useful in severe cases. In severe and disabilating cases, further immunosuppressants like cyclophosphamide, mycophenolate mofetil, or rituximab should be added to glucocorticoids and IV IG [12]. The clinical course of SS is usually self-limited, fluctuating, and monophasic. It lasts from two to four years but may be as short as six months or as long as five years in duration [13], but the exact duration in the individual patient is unpredictable. Relapses after decades, have been described. This requires monitoring for a lifelong time [12]. Although some patients recover with little or no residual disease, others are profoundly impaired with cognitive deficits, gait disturbance, and hearing loss. Usually vision is not seriously impaired [13].

A high index of suspicion, leading to correct diagnosis and early appropriate therapy may reduce the permanent sequel seen with this disease. Misdiagnosis is common. In patients in whom diagnosis and treatment are delayed permanent morbidity is higher in terms of visual loss, hearing loss, and neurologic debility. In patients in whom rapid diagnosis has led to early administration of immunosuppressive therapy, recovery can be almost complete [10].
